# A novel variant in the 3′ UTR of the *TCF4* gene likely causes Pitt-Hopkins syndrome: a case report

**DOI:** 10.1186/s13023-024-03383-8

**Published:** 2024-10-07

**Authors:** Tingting Zhao, Fan Yang, Bingbing Zhang, Yongyong Ren, Jiuzhou Yuan, Yu Wang, Hui Lu, Guangjun Yu, Jincai Feng

**Affiliations:** 1 Shanghai Engineering Research Center for Big Data in Pediatric Precision Medicine, Center for Bio-medical Informatics, Shanghai Children’s Hospital, School of Medicine, Shanghai Jiao Tong University, Shanghai, China; 2 Shanghai Institute of Medical Genetics, Shanghai Children’s Hospital, School of Medicine, Shanghai Jiao Tong University, Shanghai, China; 3 Kunming Medical University Haiyuan College, Yunnan, China; 4 SJTU-Yale Joint Center for Biostatistics and Data Science, National Center for Translational Medicine, Shanghai Jiao Tong University, Shanghai, China; 5 Department of Rehabilitation, Shanghai Children’s Hospital, School of Medicine, Shanghai Jiao Tong University, Shanghai, China; 6 Diagnosis and Treatment Center of Pitt-Hopkins Syndrome, Shanghai Children’s Hospital, School of Medicine, Shanghai Jiao Tong University, Shanghai, China

**Keywords:** Pitt-Hopkins syndrome, *TCF4*, Clinically diagnosed, Molecularly diagnosed, Whole-genome sequencing

## Abstract

**Background:**

Pitt–Hopkins syndrome (PTHS) is a rare neurodevelopmental disorder that results from variants of *TCF4* gene. PTHS follows an autosomal dominant inheritance pattern and the underlying pathological mechanisms of this disease are still unclear.

**Methods:**

Whole-genome sequencing (WGS) was conducted to screen for potential pathogenic variant in a boy highly suspected of having a genetic disorder. PCR and Sanger sequencing were used to verify the effects of the variant. Serum TCF4 levels were measured by ELISA.

**Results:**

We present a 4-year and 3-month-old Chinese boy clinically and molecularly diagnosed with PTHS. The proband experienced global development delay, and the preliminary clinical diagnosis was cerebral palsy. WGS identified a de novo heterozygous variant: c.*1A > G in the 3’UTR of the *TCF4* gene as a potential cause of his condition. The variant was verified to cause aberrant mRNA splicing by PCR and the aberrant splicing was confirmed by Sanger sequencing.

**Conclusion:**

The study identified and demonstrated the pathogenicity of a novel 3’UTR site *TCF4* variant for the first time. This research enhances understanding of pathogenetic mechanisms of PTHS and aids genetic counseling and diagnosis.

## Background

Pitt-Hopkins syndrome (PTHS, MIM #610,954) is a rare neurodevelopment disorder caused by the damaging variants in transcription factor 4 gene (*TCF4*), which encodes a member of the helix–loop–helix family of proteins widely expressed throughout the body and during neural development [1]. Diverse pathogenic variants found in the hundreds of patients with PTHS described so far include frameshift (~ 30% cases), nonsense (~ 15%), missense (~ 15% of cases) and splice-site (~ 10%) variants, as well as translocations and large deletions that encompass *TCF4* partially or entirely (~ 30%) [2]. Moreover, genome-wide association studies suggested that polymorphisms in *TCF4* are also linked with schizophrenia and other psychiatric and neurological conditions [1, 3–5].

PTHS is characterized by a range of aberrant phenotypes including distinctive facial features, delayed motor and cognitive development, possible breathing problems and/or recurrent seizures, gastrointestinal issues, and absence of speech [6–8]. However, more and more cases with mild to moderate intellectual disability have been reported in recent years [9–11]. Due to its heterogeneity and rarity, further research is needed.

Here, we report a novel variant in the 3’UTR of *TCF4* (NM_001083962.2): c.*1A > G in a Chinese boy with PTHS. The variant was identified and was confirmed to affect splicing by PCR and Sanger sequencing.

## Methods

### Patient and samples

This research involved a Chinese boy who exhibited global development delay from an early age. Chromosome microarray analysis and whole -exome sequencing (WES) were performed before, yielding negative results. Based on the clinical presentation, the clinicians highly suspected a genetic disorder and determined that further analysis with genetic sequencing was needed. Peripheral blood was collected from the proband and his parents for this purpose. Written informed consent for participation in this study and publication of the findings was obtained from the patient’s parent. This study received ethics approval from Shanghai Children’s Hospital and was conducted in accordance with the Declaration of Helsinki.

### Whole‑genome sequencing

Genomic DNA was extracted from the blood using QIAamp 96 DNA QIAcube HT Kit (QIAGEN) according to the instructions. Whole‑genome sequencing (WGS) was performed for the family to search for phenotype-associated genes on a NovaSeq 6000 (Illumina), with an average depth of approximately 33x.

### PCR and Sanger sequencing

PCR was conducted to verify the effects of the variant. The PCR primers were showed as follows:

Forward Primer: 5‘-CTGCGGGTCCGTGACATCAACGAGGCT-3’.

Reverse Primer: 5‘-GCCTGTACATACTGCTTTGCACATTC-3’.

The length of wildtype production was 552 bp. The Sanger sequencing was completed by Saihenbio.

### Estimation of serum TCF4 levels

Serum TCF4 levels were measured by the commercially available high sensitivity ELISA kits (MEIMIAN, Jiangsu, China), using Synergy2 BioTek.

## Results

### Clinical manifestations

The patient was a 4-year and 3-month-old Chinese boy at the time of last examination, with no family history of intellectual disability within three generations. The boy was the second child of a non-consanguineous pair and had a healthy old sister. Accelerated intrauterine growth was noted during approximately the 5–6 months of gestation. Additionally, the mother experienced low blood sugar during pregnancy. Delivery was unremarkable and the birth weight was 3750 g.

The child was first observed unable to sit and respond to calls when he was 6 months old. He had previously been diagnosed with cerebral palsy and received rehabilitation since the age of 13 months. By 18 months, the child could crawl on his belly and stand with support. At the age of 3 months, he still couldn’t stand or walk independently. Up to the last examination, he had not exhibited spontaneous language and shown poor cognitive development.

**Fig. 1:**
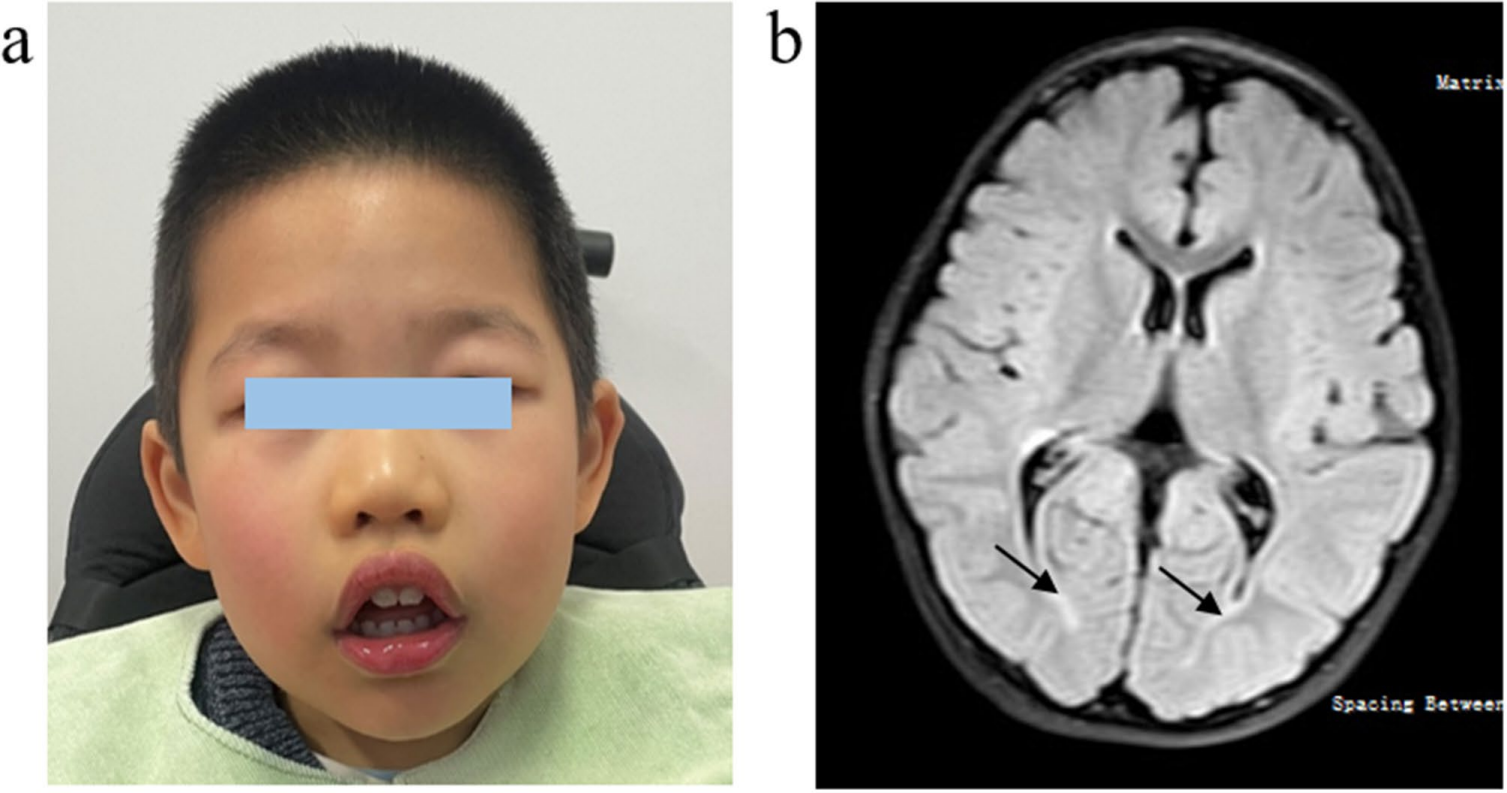
Clinical manifestations. **a** Face picture of the patient. **b** Brain MRI of the patient. T2 FLAIR showing a small abnormal signal in the bilateral posterior horn of the lateral ventricles (arrows)

The patient visited the genetic clinic of Shanghai Children’s Hospital for consultation. The proband was found to present some facial abnormalities including narrow forehead, square forehead, cupid’s bow lips, and cup-shaped ears, which are characteristic features of PTHS facial appearance (Fig. 1a). Evaluation of language, intellectual, attention/executive, memory, gross-motor/fine-motor, adaptive, and emotional/behavioral functioning revealed global impairment across all areas of functioning. Cardiac ultrasound, electroencephalogram, blood urine metabolism, and thyroid function tests yielded normal results. While the head magnetic resonance imaging (MRI) revealed a small number of abnormal signal shadows near the posterior corners of both ventricles, indicative of myelination dysplasia (Fig. 1b). He exhibited a cheerful disposition and displayed some stereotyped behaviors, such as clapping hands. Additionally, he had strabismus, occasional sighing, and a long-standing history of constipation. Allergen and food intolerance testing were conducted, revealing multiple substance allergies or intolerances.

### Genetic analysis

A heterozygous c.*1A > G variant in the 3′ UTR of *TCF4* (NM_001083962.2), one nucleotide away from the stop codon in exon 19, was detected. The variant was de novo and not found in the ExAC and gnomAD (v4.1.0) databases in the human population. SpliceAI [12] was used to predict the effect of variants on splicing, and the results were presented in Table 1. As shown, the probability of the position 5 bp downstream of TCF4 c.*1A > G being used as a new splice donor increased by 0.79, while the probability of using the original splice donor position (3 bp upstream of the variant) decreased by 0.44. This suggested that an 8 bp deletion might have occurred in the mRNA, spanning from − 3 bp to 5 bp relative to the variant. RNA Splice (https://rddc.tsinghua-gd.org/zh/ai/rna-splicer) predicted that the variant might lead to 3 kinds of abnormal splice pattern, new splice donor generating, deleting 8 bp; exon 19 skipping, deleting 141 bp; intron 10 retention, inserting 190 bp. SpliceVault [13] prediction result showed that it might lead to 8 bp deletion of mRNA in blood, fibroblasts, lymphocytes and muscle skeletal; 5 bp insertion of mRNA in fibroblasts, lymphocytes and muscle skeletal; exon 19 skipping of mRNA in lymphocytes; 69 bp insertion of mRNA in fibroblasts, lymphocytes and muscle skeletal.

**Table 1:** SpliceAI predictions A (Δ) score of 0.20 or more was considered significant. The position indicates the location of the predicted splice effect relative to the variant, measured in base pairs (bp). (+) values occur downstream of the variant, and (−) values upstream of the variant location

Gene symbol	variant	TYPE	Delta score	position
TCF4	c.*1A > G	acceptor gain	0	15
		acceptor loss	0	-3
		donor gain	0.79	5
		donor loss	0.44	-3

### Functional analysis

**Fig. 2:**
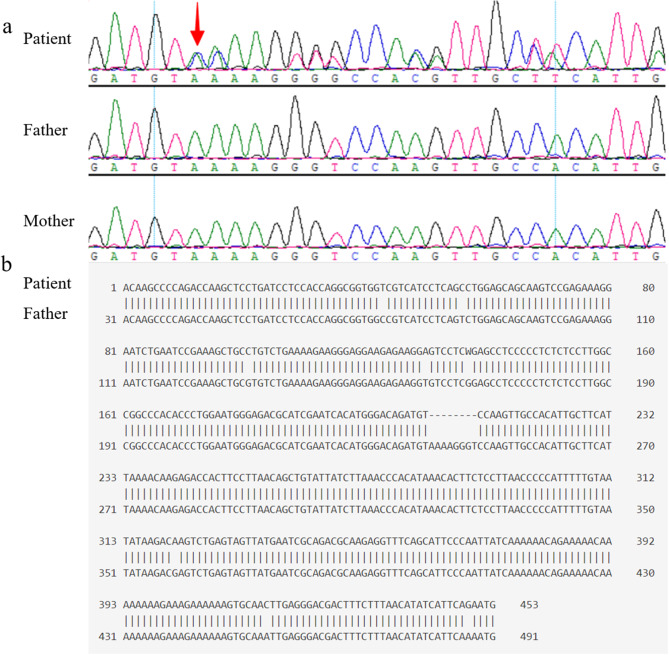
Aberrant PCR products. **a** Sanger sequencing result showed the presence of various PCR products in the patient; **b** The sequence disparities between the two distinct PCR products from the patient

Two PCR products were detected in the patient’s blood cDNA (Fig. 2a). The new transcript exhibited a deletion of ‘AAAAGGGT’ sequence, and the stop codon ‘TAA’ lost two nucleotides ‘AA’ (Fig. 2b). Consequently, the mRNA would continue translation and result in the insertion of the peptide ‘SKLPHCFIKTRDHFLNSCIILNPHKHFSLTPIFVI’ at the C-terminal of TCF4. However, there were no significant differences in serum TCF4 levels between the patient and his parents (patient: 150.330 ± 1.741 pg/ml; father: 145.596 ± 1.925 pg/ml; mother: 139.087 ± 1.288 pg/ml).

## Discussion

In this report, we described a Chinese boy with PTHS. The proband exhibited global development delay and was initially diagnosed with cerebral palsy. According to the clinical scoring rubrics proposed by the international consensus statement [2], the proband had a score of 9 including the presence of characteristic facial features, allowing for a direct clinical diagnosis of PTHS. Due to the negative WES results, WGS was conducted, identifying a heterozygous variant (c.*1A > G) in the 3’UTR of *TCF4* as a potential cause of the symptoms. The variant was subsequently validated to cause aberrant mRNA splicing by PCR and Sanger sequencing. The variant could be classified as Likely Pathogenic with the PVS1_Moderate, PS2, and PM2_Supporting evidence [14–16].

Although some gene therapies have shown promise in animal models [17], there are no effective treatments available for PTHS in clinical practice. However, accurate diagnosis can relieve emotional distress for families and facilitate better clinical management of the disease. In the case of PTHS, rehabilitation training, including physical therapy, occupational therapy and speech therapy has been implemented to address the early-onset developmental delay. Given that epilepsy occurs in approximately 37–50% of PTHS patients [2], we avoid acupuncture and electrotherapy primarily to prevent exacerbating or triggering seizures. During follow-up appointments, particular attention should be paid to the following aspects: (1) breathing anomalies; (2) gastrointestinal issues; (3) vision and hearing abnormalities, with timely interventions tailored to specific symptoms. For the constipation issue in this patient, he was instructed to avoid contact and consumption of substances that trigger intolerance and allergies. Furthermore, the Specific Carbohydrate Diet was implemented [8], leading to improvements in the patient’s constipation and mood. However, further large-scale tracking studies are required to better inform clinical practice regarding the efficacy of this intervention.

Variants in noncoding regions have been shown to disrupt the normal pre-mRNA splicing [8, 9, 18] and account for 10% of pathogenic variants [2, 19]. The detected heterozygous splice variant (c.*1A > G) ultimately led to an 8 bp deletion within the mRNA containing a stop codon, thereby causing the disruption of the protein translation termination signal and the extension of the CDS by 108 bp. This also indicated that the length of the 3’UTR was decreased. This change does not directly affect any known domains but affect all isoforms of the TCF4 gene. Another noncoding variant (c.*4 + 1G > A) in the 3’UTR of the TCF4 gene was reported in a developmental disorder study [20]. Its location was close to TCF4 c.*1A > G, but the functional study was still lacking. This also indicates that the splicing variants located near the stop codon of TCF4 may have the similar pathogenic mechanism and require further investigation. A study [19] systematically investigated the functional effects of various types of pathogenic variants, including a frameshift variant (S653Lfs*57) located in exon 20, which extends the coding region to the terminal non-coding exon 21. The study demonstrated that S653Lfs*57 results in protein degradation, aggregation, and subsequent impaired DNA binding to the E-box by the elongated TCF4 isoform [19]. However, ELISA in our study showed that the expression and stability of elongated TCF4 isoform were not affected, which was different from the findings of the previous study. An alternative explanation is that the c.*1A > G may disrupt dimer stability and/or function depending on dimerization partners and/or genomic context [1]. Additional functional analysis of the splice variant and elongated TCF4 isoform is recommended, as it may provide further insights into the pathogenic mechanism of PTHS.

## Conclusion

We utilized WGS to investigate the genetic etiology of the disease in a Chinese boy initially diagnosed with cerebral palsy. A novel heterozygous splice variant (c.*1A > G) in the 3’UTR of the *TCF4* gene was identified. The proband was further clinically and molecularly diagnosed with PTHS. To our knowledge, this is the first functional annotation of a splicing variant in the 3’UTR of *TCF4*. With continued research of the *TCF4* gene and increased awareness of PTHS, we hope to build a more comprehensive understanding of the *TCF4* gene spectrum and PTHS.

## Data Availability

The data presented in this study are available on request. The data are not publicly available due to privacy restrictions.

## Abbreviations

PTHS: Pitt-Hopkins syndrome
TCF4: transcription factor 4
WES: whole -exome sequencing
WGS: whole-genome sequencing
MRI: magnetic resonance imaging
